# Trends in the knowledge, attitudes and practices of travel risk groups towards prevention of malaria: results from the Dutch Schiphol Airport Survey 2002 to 2009

**DOI:** 10.1186/1475-2875-11-179

**Published:** 2012-05-29

**Authors:** Perry JJ van Genderen, Pieter PAM van Thiel, Paul GH Mulder, David Overbosch

**Affiliations:** 1 Harbor Hospital and Institute for Tropical Diseases, Haringvliet 72, 3011 TG, Rotterdam, The Netherlands; 2 Travel Clinic Havenziekenhuis, Rotterdam, The Netherlands; 3 Division of Infectious Diseases and Tropical Medicine and Center for Infection and Immunity Academic Medical Center, University of Amsterdam, Amsterdam, The Netherlands; 4 Department of Biostatistics, Erasmus University Hospital, Rotterdam, The Netherlands

**Keywords:** Malaria, Traveller, Travel, Risk, Knowledge, Attitude, Practice, KAP, VFR, Business, Solo, Single, Elderly

## Abstract

**Background:**

Previous studies investigating the travellers’ knowledge, attitudes and practices (KAP) profile indicated an important educational need among those travelling to risk destinations. Initiatives to improve such education should target all groups of travellers, including business travellers, those visiting friends and relatives (VFRs), and elderly travellers.

**Methods:**

In the years 2002 to 2009, a questionnaire-based survey was conducted at the Dutch Schiphol Airport with the aim to study trends in KAP of travel risk groups towards prevention of malaria. The risk groups last-minute travellers, solo-travellers, business travellers, VFRs and elderly travellers were specifically studied.

**Results:**

A total of 3,045 respondents were included in the survey. Travellers to destinations with a high risk for malaria had significantly more accurate risk perceptions (knowledge) than travellers to low-risk destinations. The relative risk for malaria in travellers to high-risk destinations was probably mitigated by higher protection rates against malaria as compared with travellers to low risk destinations. There were no significant differences in intended risk-taking behaviour. Trend analyses showed a significant change over time in attitude towards more risk-avoiding behaviour and towards higher protection rates against malaria in travellers to high-risk destinations. The KAP profile of last-minute travellers substantially increased their relative risk for malaria, which contrasts to the slight increase in relative risk of solo travellers, business travellers and VFRs for malaria.

**Conclusions:**

The results of this sequential cohort survey in Dutch travellers suggest an annual 1.8% increase in protection rates against malaria coinciding with an annual 2.5% decrease in intended risk-seeking behaviour. This improvement may reflect the continuous efforts of travel health advice providers to create awareness and to propagate safe and healthy travel. The KAP profile of last-minute travellers, in particular, substantially increased their relative risk for malaria, underlining the continuous need for personal protective measures and malaria chemoprophylaxis for this risk group.

## Background

In 2008, a cluster of 56 European tourist travellers returned from The Gambia with *Plasmodium falciparum* malaria. Three of them died. The common denominator of all patients was that they booked a last-minute vacation in The Gambia and did not use adequate malaria chemoprophylaxis or used it wrongly [1]. Even though recent trend analysis in the Netherlands showed a reassuringly significant decline in the number of cases with imported malaria, the steadily increasing number of Dutch travellers to malaria endemic region not using malaria chemoprophylaxis remains worrisome [2]. An improved effort is needed to increase awareness and protection among this growing number of unprotected travellers to malaria endemic regions.

The risk of a traveller for contracting a travel-related infectious disease like malaria is not only depending on the destination of travel and planned activities, but also on the traveller’s personal risk profile. The main determinants of the traveller’s personal risk profile are usually presented as the knowledge, attitude and practice (KAP) of a traveller towards prevention of travel-related infectious disease. In these studies knowledge is usually defined as an accurate risk perception, whereas attitude is commonly defined as either intended risk-seeking or risk-avoiding behaviour. Finally, practice is defined as the rate of protection rate against a certain travel-related infectious disease.

In the years 2002–2003 the European Travel Health Advisory Board conducted a cross-sectional pilot survey in several European airports including the Dutch Schiphol Airport to evaluate current travel health knowledge, attitudes and practices (KAP) towards prevention of hepatitis A, hepatitis B and malaria and to determine where travellers going to developing countries obtain travel health information, what information they receive, and what preventive travel health measures they adopt [3,4]. The results of these studies also demonstrated an important educational need among those travelling to risk destinations. Initiatives to improve such education should target all groups of travellers. In the Netherlands, a similar survey has been done each year between 2002 and 2009 (except for the year 2006), giving a unique opportunity to study trends in KAP of travellers towards prevention of travel-related infectious diseases. In the present study, the findings regarding these trends towards prevention of malaria are reported with a special focus on the risk groups last-minute travellers, solo-travellers, business travellers, travellers visiting friends and relatives (VFR), as well as elderly travellers.

## Methods

### Questionnaires and survey

The survey was conducted as previously described [3,4]. In brief, self-administered, anonymous questionnaires were randomly distributed at the departure gate of Schiphol Airport, Amsterdam, The Netherlands, while passengers were waiting to board. Intercontinental flights to destinations with an intermediate or high risk for hepatitis A, hepatitis B or malaria were preferably selected. The survey was always done in the same period of the year, namely the months October or November. Travellers participated on a voluntary basis; no incentive was provided, except for a leaflet with information on hepatitis A, hepatitis B and malaria. Trained interviewers were present to distribute the questionnaires, to answer questions if necessary and to check the completeness of the responses collected. When possible, these interviewers copied the information from the travellers’ vaccination records. Travellers were allowed to participate if they were 18 years of age or older, and able to fully understand the language of the questionnaires. They also had to be resident in the Netherlands; thus, nationals of a developing country were only asked to participate if they were actually living in the Netherlands. These criteria were checked by the interviewers when distributing the forms. Afterwards, completed questionnaires from travellers who did not meet all the inclusion criteria were either excluded by the interviewers or rejected from the final analysis.

Two kinds of questionnaires were distributed among the participants, depending on the precise destination. The malaria questionnaire (Q-mal) focused on malaria and its prevention and treatment and these questionnaires were distributed only to travellers with destinations in or close to malaria-endemic areas. The vaccine questionnaire (Q-vacc) targeted the vaccine-preventable travel-related diseases hepatitis A and B. Both questionnaires had a common part on personal characteristics (age, gender, nationality, residence, profession), on information regarding the travel (destination, duration, purpose, travel companions) and its preparation, and on the travellers’ perception of the risk of malaria, hepatitis A and hepatitis B at their destination. However, since most malaria-endemic countries also carry a high risk for hepatitis A and B, the Q-mal questionnaire also contained several items dealing with the KAP towards prevention of hepatitis A and B.

### Definitions of risk groups

Respondents with an age over 60 years were arbitrarily classified as elderly travellers. Solo travellers were defined as those travellers who travelled alone. Business travellers were defined as those travellers who specifically stated that their main purpose for travel was business-related. Last-minute travellers were defined as those travellers who did not seek pre-travel health advice or sought it only within two weeks before departure. Respondents who specifically stated that their main purpose for travel was to visit friends and relatives were considered VFRs.

### Determination of KAP profile on malaria

Knowledge of malaria was determined by comparison of the risk for malaria as perceived by the traveller with the actual risk for malaria, as described [5]. To that end, all destinations (including those in malaria-endemic countries) were rated as low or high-risk destination for malaria based on maps published by the Centers for Disease Control, Atlanta, USA [6]. Destinations rated as low-risk also comprised destinations without any risk of malaria (no-risk area). For each subject the accuracy (correct risk perception) was expressed as 0 or 1, with 1 assigned to a subject if his (her) knowledge about risk was compatible with the official risk rating of the destination. To determine the attitude (intended risk taking or risk avoiding behaviour) of participants towards prevention of malaria, all participants were asked if they were planning to: (1.) cover their arms and legs when going outside; (2.) use of an insect repellant on uncovered skin; (3.) keep the doors and windows closed; (4.) sleep under a bed net and (5.) stay in air-conditioned surroundings. Each affirmative answer was scored with 1 point whereas a negation was scored with 0 points. The final attitude score towards prevention of malaria was obtained as the sum of the separate answer scores and could therefore range from 0 to 5; for convenience, the score was transformed to a 0–100 scale with the maximal protective attitude score set at 100. To have an indication of their practice (protection rate) towards prevention of malaria travellers were asked whether they had packed personal protective measures like insect repellents, bed nets and malaria chemoprophylaxis for this trip. Protection rate was expressed as a weighted sum of use of insect repellent (1 point), use of bed net (2 points) and use of malaria chemoprophylaxis (3 points). The practice sum score could, therefore, range from 0 to 6; for convenience, the score was transformed to a 0–100 scale with the maximal practice score set at 100. In order to estimate the impact of KAP of the travel risk group of interest on relative risk for malaria, a composite risk estimate was constructed by summing up the effects of the separate determinants. To that end, it was assumed that either a poor risk perception, intended risk-seeking behaviour or poor protection rates led to an equal increase in relative risk for malaria.

### Statistical analysis

Several statistical analyses were made between travellers to high- and low-risk destinations: on one hand the so-called “between malaria risk destinations” analysis: *e.g*. the comparison of VFRs travelling to a destination with a high malaria risk *vs* VFRs travelling to a destination with a low malaria risk and on the other hand the so-called “within malaria risk destination” analyses: *e.g.* the comparison of solo-travellers to destinations with a high malaria risk *vs* the remaining (non-solo) travellers to this high malaria risk destination. To that end firstly differences in the predefined risk factor distributions between the two different risk destinations were tested using multiple logistic regression analyses, adjusted for subpopulation (maximally 14 subpopulations: two kinds of questionnaires by 7 interview years). Next, similar logistic regression analyses with adjustment for subpopulation were done for testing differences in risk (e.g., yes *vs* no VFR as independent variable) between the two knowledge groups (accurate risk perception y/n as dependent variable), allowing separate tests within low and within high risk destinations through entering the appropriate interaction terms into the models. The dependency of the attitude and practice scores on the risk factors was analyzed using multiple linear regression analyses, modeled similarly to the above mentioned logistic regression analyses. Those regression analyses also allow testing differences between the two risk destination groups in knowledge, attitude and practice within specific risk groups. Finally, it was tested by entering the appropriate interaction terms in the multiple logistic and linear regression models if the strength of the effect of the predetermined risk factors on knowledge, attitude and practice showed a significant time trend over the years 2002 to 2009 within low as well as within high risk destinations.

## Results

### Study population

**Figure 1:**
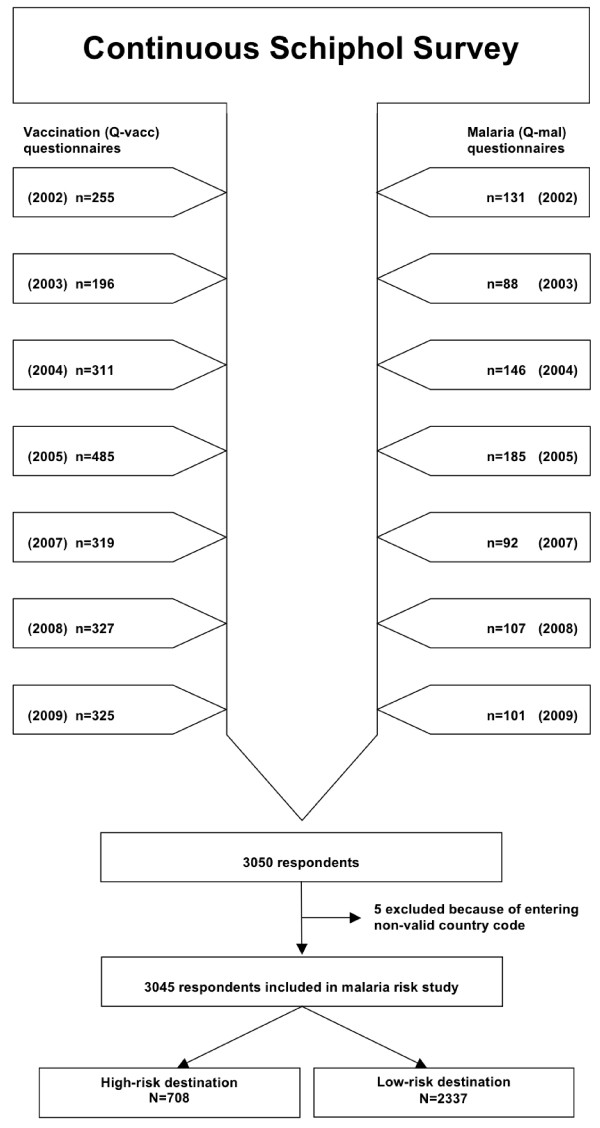
**Flowchart of the Dutch Schiphol Airport Survey.** The yearly inclusions of respondents of the malaria questionnaires (Q-mal) and vaccination questionnaires (Q-vacc) in the study are shown as well as reasons for exclusion.

Across all seven years in the period from 2002 to 2009 (except year 2006), a total of 3,050 questionnaires were received, of which 3,045 fulfilled the entry criteria and were included in the analysis (Figure 1). Of the 3,045 respondents, 708 respondents travelled to destinations with a high risk for malaria. The remaining 2,337 respondents travelled to a low malaria risk destination. The general characteristics of all respondents, grouped by malaria risk in high and low risk destinations, are shown in Table 1. Overall, 46.4% of responders were female and 53.6% were male. Almost 71% of the travellers to high malaria risk destinations had malaria chemoprophylaxis (proguanil 3.0%; mefloquine 6.6%; doxycycline 1.0%; atovaquone/proguanil 47.0%; other 12.7%) with them and had packed insect repellents. Of the 1,324 travellers to a destination without malaria risk, 12 (0.9%) had packed malaria chemoprophylaxis.

**Table 1:** General characteristics of 3045 respondents in relation to the malaria risk profile of their destination

		**High risk destination**		**Low-risk destination**		***P* - value**^**2**^
		N	%	N	%	
		708	23.3	2337	76.7	
**Sex**						
	*Male*	358	50.6^**1**^	1265	54.1^**1**^	0.613
	*Female*	345	48.7	1058	45.3	
**Age**						
	*Age > 60 yrs*	128	18.1	311	13.3	0.388
**Travel duration**						
	*< 7 days*	139	19.6	561	24.0	0.000
	*8–14 days*	242	34.2	926	39.6	
	*15–28 days*	167	23.6	591	25.3	
	*> 28 days*	87	12.3	190	8.1	
**Travel health preparation**						
**Pre-travel information**						0.014
	*No*	108	15.3	943	40.4	
	*Yes*	600	84.7	1394	59.6	
**Time frame information-departure**						0.005
	*< 7 days*	79	11.2	152	6.5	
	*8–14 days*	115	16.2	199	8.5	
	*15–28 days*	159	22.5	342	14.6	
	*> 28 days*	247	34.9	701	30.0	
**Purpose for travel**						0.000
	*Tourist*	381	53.8	1546	66.2	
	*Business*	102	14.4	351	15.0	
	*VFR*	154	21.8	367	15.7	
	*Missionary/volunteer*	43	6.1	22	0.9	
	*Research*	13	1.8	8	0.3	
	*Other*	10	1.4	21	0.9	
**Travel profile**						0.000
	*Solo traveller*	209	29.5	660	28.2	
	*Travel with spouse*	286	40.4	908	38.9	
	*Travel with children*	68	9.6	267	11.4	
	*Travel with group*	47	6.6	106	4.5	
	*Travel with friends*	43	6.1	155	6.6	
	*Other*	29	4.1	90	3.9	
**Preventive measures**						
	*Insectrepellants*	501	70.8	184	7.9	0.954
	*Bednet*	138	19.5	29	1.2	0.007
	*Chemoprophylaxis*	498	70.3	99	4.2	0.000
**Travel experience (intercontinental) in last 5 years**						0.971
	*No*	112	15.8	383	16.4	
	*Yes*	441	62.3	1440	61.6	
**Most popular destinations**						**Most popular destinations**
	*Gambia*	373	52.7	428	18.3	*Turkey*
	*Surinam*	101	14.3	418	17.9	*Egypt*
	*Ghana*	66	9.3	180	7.7	*Mexico*
	*Nigeria*	49	6.9	176	7.5	*Thailand*
	*Uganda*	31	4.4	172	7.4	*China*

Legend: ^1^ = all data are given as a percentage of either the total number of respondents to high risk destinations (*i.e*., n = 708) or as a percentage of the total number of respondents to low risk destinations (*i.e*., n = 2337); ^2^ = *P*-value for comparison of high-risk destinations *vs* low risk destinations, adjusted for year and kind of questionnaire.

### Travel profile

For 20.8% of the travellers since 2004, it was their first trip to a developing country (there was no first-trip-item in the questionnaires of 2002 and 2003). Overall, 63.9% indicated tourism as their purpose of travel. One in five to six responders were visiting friends and relatives, business travellers accounted for 15.0% . Few responders travelled for missionary reasons or for voluntary missions (2.2%), for purpose of research or education (0.7%) or for other reasons (1.0%). Many travellers (41.6%) were accompanied by their partner or spouse; 869 persons (30.3%) were travelling alone, 6.9% with friends, 11.7% with children.

Travellers to high malaria risk destinations planned to stay significantly longer on their destination than travellers to low-risk destinations (*p* < 0.001) and obtained pre-travel health advice more frequently prior to departure (*p* < 0.001). Overall, 24.1% went abroad for 1 to 7 days, 40.2% for 8 to 14 days, 26.1% for 15 to 28 days, and 9.5% for more than 28 days. The Gambia was the most common high-risk destination (52.7%), followed by Surinam (14.3%) and Ghana (9.3%) whereas among the low risk destinations Turkey (18.3%) was the most common destination, followed by the Egypt (17.9%) and Mexico (7.7%) (Table 1).

### Travel health preparations

The majority of travellers (65.5%) had sought health information about their destination prior to departure. This was done more than one month before leaving by 47.5% of the responders; 25.1% started preparing two weeks to one month before departure, 15.7% did so one to two weeks in advance, and 11.6% did so less than one week before leaving.

Of those who had not sought health information, the majority stated that they already knew what to do. The most common sources since 2004 for travel health advice to high risk destinations were the travel clinic or public health service (27.8%) followed by general practitioner (GP) or family doctor in 11.8% of the respondents. For low risk destinations the travel clinic or public health service was consulted more frequently in 51.0% of the respondents, whereas the GP or family doctor was consulted in 10.2% of the cases (*p* = 0.005). In the 2002- and 2003-questionnaires there was no item concerning source of advice. There was a significant positive trend over the years in the proportion of travellers to high-risk destinations seeking travel health advice (*p* = 0.002).

### Travel risk groups

The group of elderly travellers comprised 439 respondents. Of them, 128 (29.2%) travelled to a high-risk destination. The group of last-minute travellers comprised of 545 respondents; 194 (35.6%) of them travelled to a high-risk destination. Of all respondents, 869 respondents travelled alone and were classified as solo-travellers; 209 (24.1%) of them travelled to a high-risk destination. The group of business travellers consisted of 453 individuals of whom 102 (22.5%) travelled to destinations rated as a high-risk destination. The group of VFRs consisted of 521 respondents; 390 (29.6%) of them travelled to a high-risk destination (Table 1).

### KAP of malaria: analysis of risk groups

#### Elderly travellers

Elderly and younger aged travellers did not significantly differ in visiting high risk destinations (Table 1: *p* = 0.388, adjusted for subpopulation). Elderly travellers to either high risk or low risk destinations did not seek pre-travel information significantly more often than younger aged travellers to the same risk destination (*p* = 0.158 for low risk and *p* = 0.900 for high risk destinations, adjusted for subpopulation). The knowledge, attitude and practice of elderly travellers to high-risk destinations was comparable to that of younger travellers to same risk destinations (Tables 2 and 3). As a consequence, as shown in Table 4, the relative risks of elderly travellers for malaria was comparable to that of younger travellers.

**Table 2:** Knowledge, attitude and practice of travel risk groups to destinations with a high risk for malaria

**Knowledge**^**1**^	**High risk destinations**					
	**# cases**	**# total**	**%**	***p *- value**	**95% CI mean**	***p *- value**
				**(within high risk group)**^**a**^**)**		**(high vs low risk group**^**c**^**)**
*Overall*	516	708	72.9	n.a.	69.6–76.2	0.000
*Elderly traveller*	92	128	71.9	0.466	64.1–79.7	0.000
*Solo-traveller*	186	244	76.2	0.075	70.9–81.6	0.000
*Business-traveller*	85	105	81.0	0.079	73.4–88.5	0.000
*Last-minute traveller*	206	302	68.2	0.041	63.0–73.5	0.000
*VFR*	114	154	74.0	0.298	67.1–81.0	0.000
**Attitude**^**2**^	**# cases**	**mean**	**sd**	***p *- value**	**95% CI mean**	***p *- value**
				**(within high risk group**^**a**^**)**		**(high vs low risk group**^**c**^**)**
*Overall*	517	72.0	24.6	n.a.	69.8–74.2	0.491
*Elderly traveller*	99	69.9	26.9	0.374	64.5–75.3	0.896
*Solo-traveller*	174	68.6	25.8	0.053	64.7–72.5	0.042
*Business-traveller*	90	70.2	25.1	0.316	64.9–75.5	0.755
*Last-minute traveller*	211	67.5	25.4	0.005	64.0–71.0	0.207
*VFR*	80	66.8	27.4	0.062	60.7–72.9	0.364
**Practice**^**3**^	**# cases**	**mean**	**sd**	***p *- value**	**95% CI mean**	***p *- value**
				**(within high risk group**^**a**^**)**		**(high vs low risk group**^**c**^**)**
*Overall*	610	61.9	27.7	n.a.	59.7–64.1	0.000
*Elderly traveller*	105	61.6	26.4	0.151	56.4–66.8	0.009
*Solo-traveller*	197	54.5	31.6	0.000	50.0–59.0	0.000
*Business-traveller*	94	51.8	31.7	0.000	45.3–58.3	0.016
*Last-minute traveller*	249	57.4	29.2	0.001	53.7–61.1	0.000
*VFR*	112	53.9	31.9	0.004	47.9–59.9	0.000

Legend: ^1^ = knowledge was defined as a percentage of maximal accurate risk perception (0–100% scale); ^2^ = attitude was defined as a percentage of maximal risk behaviour (0–100% scale); ^3^ = practice was scored only in malaria questionnaire (Q-mal) as a percentage of maximal protection. ^a^ = *p*-value of comparison of a given risk group vs the remainder of travellers to a high risk destination; ^c^ = *P*-value of comparison of a given risk group to a high risk destination vs the same risk group to a low risk destination; n.a. = not applicable; n.s. = not significant; Last-minute = information sought: none or within 14 days before departure.

**Table 3:** Knowledge, attitude and practice of travel risk groups to destinations with a low risk for malaria

**Knowledge**^**1**^	**Low risk destinations**					
	**# cases**	**# total**	**%**	***p *- value**	**95% CI mean**	***p *- value**
				**(within low risk group**^**b**^**)**		**(high vs low risk group**^**c**^**)**
*Overall*	1021	2337	43.7	n.a.	41.7–45.7	0.000
*Elderly traveller*	114	311	36.7	0.006	31.3–42.0	0.000
*Solo-traveller*	332	766	43.3	0.661	39.8–46.9	0.000
*Business-traveller*	150	352	42.6	0.829	37.4–47.8	0.000
*Last-minute traveller*	532	1294	41.1	0.019	38.4–43.8	0.000
*VFR*	156	367	42.5	0.437	37.4–47.6	0.000
**Attitude**^**2**^	**# cases**	**mean**	**sd**	***p *- value**	**95% CI mean**	***p*-value**
				**(within low risk group**^**b**^**)**		**(high vs low risk group**^**c**^**)**
*Overall*	189	71.7	25.7	n.a.	68.0–75.4	0.491
*Elderly traveller*	36	71.1	21.6	0.760	63.9–78.3	0.896
*Solo-traveller*	41	61.0	33.5	0.002	50.5–71.5	0.042
*Business-traveller*	8	65.0	38.2	0.667	38.0–92.0	0.755
*Last-minute traveller*	72	72.8	24.7	0.343	67.0–78.6	0.207
*VFR*	25	64.8	30.2	0.057	52.7–76.9	0.364
**Practice**^**3**^	**# cases**	**mean**	**sd**	***p *- value**	**95% CI mean**	***p* - value**
				**(within low risk group**^**b**^**)**		**(high vs low risk group**^**c**^**)**
*Overall*	228	39.4	32.2	n.a.	35.1–43.7	0.000
*Elderly traveller*	41	46.3	28.3	0.371	37.5–55.1	0.009
*Solo-traveller*	47	32.3	33.6	0.056	22.5–42.1	0.000
*Business-traveller*	10	28.3	30.5	0.173	9.0–47.6	0.016
*Last-minute traveller*	84	38.5	32.9	0.844	31.3–45.7	0.000
*VFR*	35	29.5	33.6	0.108	18.1–40.9	0.000

Legend: ^1^ = knowledge was defined as a percentage of maximal accurate risk perception (0–100% scale); ^2^ = attitude was defined as a percentage of maximal risk behaviour (0–100% scale); ^3^ = practice was scored only in malaria questionnaire (Q-mal) as a percentage of maximal protection. ^b^ = *P*-value of comparison of a given risk group vs the remainder of travellers to a low risk destination; ^c^ = *P*-value of comparison of a given risk group to a high risk destination vs the same risk group to a low risk destination; n.a. = not applicable; n.s. = not significant; Last-minute = information sought: none or within 14 days before departure.

**Table 4:** Estimates of the aggregate impact of the knowledge, attitude and practice (KAP) of travel risk groups on their relative risk of malaria

**Destinations with a high risk of malaria**				
**Risk group**	**Knowledge**	**Attitude**	**Practice**	**Impact on relative risk of malaria**
*Elderly traveller*	No effect on risk	No effect on risk	No effect on risk	**No effect on risk**
*Solo traveller*	No effect on risk	No effect on risk	Increase in risk	**Slight increase in risk**
*Business traveller*	No effect on risk	No effect on risk	Increase in risk	**Slight increase in risk**
*Last-minute traveller*	Increase in risk	Increase in risk	Increase in risk	**Substantial increase in risk**
*VFR*	No effect on risk	No effect on risk	Increase in risk	**Slight increase in risk**
**Destinations with a low risk of malaria**				
**Risk group**	**Knowledge**	**Attitude**	**Practice**	**Impact on relative risk of malaria**
*Elderly traveller*	Increase in risk	No effect on risk	No effect on risk	**Slight increase in risk**
*Solo traveller*	No effect on risk	Increase in risk	No effect on risk	**Slight increase in risk**
*Business traveller*	No effect on risk	No effect on risk	No effect on risk	**No effect on risk**
*Last-minute traveller*	Increase in risk	No effect on risk	No effect on risk	**Slight increase in risk**
*VFR*	No effect on risk	No effect on risk	No effect on risk	**No effect on risk**

#### Solo travellers

Solo-travellers travelled to high-risk destinations more often than non-solo travellers (*p* < 0.0005, adjusted for subpopulation). Solo-travellers to either high (*p* = 0.001, adjusted for subpopulation) or low-risk destinations (*p* < 0.0005, adjusted for subpopulation) had less preparation for their travel than non-solo travellers to the same risk destination. The risk perception and intended risk-taking behaviour of solo-travellers to high-risk destinations was comparable to that of non-solo travellers (Tables 2 and 3). Solo-travellers had significantly lower protection rates than non-solo travellers to high-risk destinations (Tables 2 and 3), suggesting that the KAP of solo-travellers resulted in a slight increase in relative risk for malaria (Table 4).

#### Business travellers

Business travellers to either high (*p* = 0.029, adjusted for subpopulation) or low-risk destinations (*p* < 0.0005, adjusted for subpopulation) less frequently sought travel health advice than non-business travellers. Business travellers travelled more frequently to high-risk destinations than non-business travellers (*p* = 0.001, adjusted for subpopulation). Business travellers to high-risk destinations had comparable knowledge and intended risk-taking attitude as non-business travellers but had significantly lower protection rates against malaria (Tables 2 and 3). As a consequence, the KAP of business travellers to high-risk destinations slightly increased their relative risk for malaria (Table 4).

#### Last-minute travellers

Last-minute travellers did not significantly differ from regular travelers in visiting high malaria risk destinations (*p* = 0.575, adjusted for subpopulation) and had comparable travel health preparation in comparison to regular travellers (high-risk destinations *p* = 0.199; low-risk destinations, *p* = 0.111). The risk perception and protection rates of last-minute travellers to high-risk destinations was significantly lower than that of regular travellers (Tables 2 and 3). In addition, last-minute travellers to high-risk destinations had less intended risk-avoiding behaviour than regular travellers. As a consequence, their KAP substantially increased their relative risk for malaria (Table 4).

#### Visiting friends and relatives

VFRs sought less frequently travel health advice than non-VFR travellers (high risk destinations *p* = 0.031; low risk destinations *p* < 0.0005). VFRs travelled more commonly to high-risk destinations than non-VFR travellers (*p* < 0.0005, adjusted for subpopulation) (Table 1). The knowledge and attitude of VFRs towards prevention of malaria was comparable to that of non-VFR travellers to high-risk destinations. However, their protection rates were significantly lower (Tables 2 and 3). As a consequence, the KAP profile of VFRs slightly increased their relative risk for malaria (Table 4).

### Trends in KAP of travellers towards prevention of malaria

#### Knowledge (accurate risk perception)

Over the years there were no significant trends in traveller’s knowledge, defined as an accurate risk perception of malaria, neither for the group as a whole nor for the pre-defined risk groups. Thus, there were no significant trends over the years in the knowledge of travellers to either low or high-risk destinations.

#### Attitude (intended risk-avoiding behaviour)

In contrast to the trend in knowledge towards prevention of malaria, in both travellers to high and low-risk destinations a significant trend could be established favouring a more intended risk-avoiding attitude. In travellers to high-risk destinations trend analysis showed an annual 2.5 percent increase (95% Confidence Interval 1.4 to 3.5; *p* < 0.0005) towards intended risk-avoiding behaviour; in travellers to low-risk destinations the increase was 2.1 percent (95% CI 0.4 to 3.8; *p* = 0.017). In none of the predefined risk group a trend change could be demonstrated, except for elderly travellers to low-risk destination, in whom an annual increase of 6.1 points (95% CI 1.1 to 11.1; *p* = 0.018) towards a more risk-taking attitude was found.

#### Practice (protection rate)

There were no significant trends over the years in use of insect repellents, bed nets or malaria chemoprophylaxis in travellers to either high or low risk destinations. However, a significant trend in protection rates of travellers to high-risk destinations was observed which was paralleled by a mean annual 1.8 percent increase (95% CI 0.8–2.7; *p* = 0.001) in protection. With regard to risk groups, also in VFRs to high-risk destinations a mean annual 2.5 percent increase in protection could be established (95% CI 0.02–5.02; *p* = 0.049). Of the risk groups travelling to low-risk destinations, annual increases in protection rates were established for solo travellers (mean 2.9 percent increase; 95% CI 0.8–5.1; *p* = 0.008) and for last-minute travellers (mean 4.2 percent increase; 95% CI 0.5–8.0; *p* = 0.028).

## Discussion

The results of the European Airport Survey demonstrated an important educational need among those travelling to risk destinations and it was suggested that travel health advice providers should continue their efforts to make travellers comply with the recommended travel health advice, especially risk groups [3,4]. The present study provides in-depth feedback on these efforts towards prevention of malaria by analysing the trends in KAP of Dutch travellers, including those belonging to a certain risk group, over an 8-year observation period. As might be expected for travellers to destinations with a high risk for malaria, they had significantly more knowledge towards prevention of malaria and were better protected against malaria than travellers to low-risk destinations. Interestingly, a significant increase in the proportion of travellers to high-risk destinations seeking travel health advice over the years was noted, which was paralleled by a significant increase in protection rates towards malaria. A plausible explanation for the higher protection rates against malaria may be that travellers to high-risk destinations who seek travel health advice are advised to use personal protective measures and malaria chemoprophylaxis without exemption opposed to travellers to low risk destinations. Further, trend analyses also indicated that – in general terms – travellers to high-risk destinations also had significant improvement over time in intended risk-avoiding attitude as well as actual protection rates against malaria (but not in knowledge of malaria), which may reflect these continuous efforts of travel health advice providers to propagate safe and healthy travel. It may also indirectly reflect an increasing awareness of Dutch travellers for the need for proper protective measures against travel-related diseases like malaria, but without asking for a detailed knowledge of these diseases.

However, the situation is still far from ideal given the finding that only approximately 70% of the travellers to high-risk destinations had actually packed malaria chemoprophylaxis and insect repellants, leaving the remaining 30% of the travelers to high-risk destinations unprotected and conceivably at an increased relative risk for contracting malaria at their travel destination. In addition, the findings of the KAP of the travel risk groups towards prevention of malaria may also raise concern. Even though travellers belonging to a certain risk group are usually among those with the highest risk profile for acquiring a travel-related disease, the current findings allow a modification of this risk profile by implementing the impact of their KAP on their relative risk for malaria. The current findings indicate that last-minute travellers to high-risk destinations are among the travellers with the worst risk profile towards prevention of malaria. These findings may - at least to a lesser extent - also apply for solo-, business travellers and VFRs to high-risk destinations.

When focusing on last-minute travellers, it is interesting to note that a significant number of respondents was travelling to The Gambia. Vacations to The Gambia are usually marketed as attractive last-minute ‘winter sun’ alternatives for the Canary Islands, Portugal or Spain. However, being located in West-Africa, travel to The Gambia requires not only proof of protection against yellow fever but has also strict indications for malaria chemoprophylaxis throughout the year. In addition, many travel brochures and booking agencies underexpose the need for malaria prophylaxis and proper travel health advice [7]. As a consequence, travellers to The Gambia are at an increased risk for contracting malaria because of this lack of awareness and prophylactic measures. In fact, during the study period in which the Dutch Schiphol Airport surveys took place, clusters of imported malaria cases in travellers returning from the Gambia were described in several European countries, including the Netherlands [1,8,9]. Last-minute booking, not seeking or adhering to travel health advice and not taking any or using inappropriate malaria chemoprophylaxis and a high case-fatality rate were the common denominators among these cases, stressing the need for proper preventive measures and increased awareness of the potential life-threatening dangers associated with travel to West-Africa for this group of travellers.

### Limitations

Questionnaire-based surveys may have some drawbacks, which may limit the generalizability of the current findings. For instance, this study was designed to study the KAP of travellers to destinations with a high or lower risk for malaria and all destinations were selected to meet this requirement. The destinations were not randomly selected from all available risk destinations. Further, the survey was always done in the months October and November of each year, which may have introduced a selection bias since people who travel at this time of year may differ from people who travel during summer vacation. Moreover, one could argue that the traveller’s KAP profile including those belonging to risk groups may be influenced by their prior travel experience. To specifically address this potential confounder, all questionnaires since 2004 contained questions elaborating on this item but no significant differences in prior travel experience were found between travellers to high or low-to-intermediate risk destinations. Lastly, not all respondents belonged mutually exclusive to one risk group; this may limit the effect attributed to a certain characteristic of a risk profile.

In conclusion, the results of this questionnaire-based survey suggest that protection rates of Dutch travellers against malaria increase every year in concert with an annual reduction in intended risk-seeking behaviour. Last-minute travellers to high-risk destinations were identified as the risk group with the highest increase in relative risk for malaria underlining the continuous need for education, proper personal protective measures and malaria chemoprophylaxis of this travel risk group.

## Competing interests

PJJVG and DO received speaker’s fee from GlaxoSmithKline as well as reimbursements for attending symposia. The other authors declare that they have no competing interests.

## Authors’ contributions

PJJVG drafted the manuscript and participated in the design of the study and analysis of the data. PPAMVT conceived of the study and participated in its coordination, and revised the manuscript. PGHM performed the statistical analysis and revised the manuscript. DO conceived of the study and participated in its coordination, and revised the manuscript. All authors read and approved the final manuscript.
